# Design of Plant Protection UAV Variable Spray System Based on Neural Networks

**DOI:** 10.3390/s19051112

**Published:** 2019-03-05

**Authors:** Sheng Wen, Quanyong Zhang, Xuanchun Yin, Yubin Lan, Jiantao Zhang, Yufeng Ge

**Affiliations:** 1 South China Agricultural University Engineering Foundation Teaching and Training Center, Guangzhou 510642, China; vincen@scau.edu.cn; 2 National Center for International Collaboration Research on Precision Agriculture Aviation Pesticides Spraying Technology, Guangzhou 510642, China; qy_zhang@stu.scau.edu.cn (Q.Z.); ylan@scau.edu.cn (Y.L.); zhangjiantao@yeah.net (J.Z.); 3 Engineering College of South China Agricultural University, Guangzhou 510642, China; 4 Mathematics and Informatics College of South China Agricultural University, Guangzhou 510642, China; 5 Biological Systems Engineering college of University of Nebraska-Lincolin, Lincoln, NE 68583, USA; yge2@unl.edu

**Keywords:** UAV, BP neural network, droplet deposition, variable spray

## Abstract

Recently, unmanned aerial vehicles (UAVs) have rapidly emerged as a new technology in the fields of plant protection and pest control in China. Based on existing variable spray research, a plant protection UAV variable spray system integrating neural network based decision making is designed. Using the existing data on plant protection UAV operations, combined with artificial neural network (ANN) technology, an error back propagation (BP) neural network model between the factors affecting droplet deposition is trained. The factors affecting droplet deposition include ambient temperature, ambient humidity, wind speed, flight speed, flight altitude, propeller pitch, nozzles pitch and prescription value. Subsequently, the BP neural network model is combined with variable rate spray control for plant protection UAVs, and real-time information is collected by multi-sensor. The deposition rate is determined by the neural network model, and the flow rate of the spray system is regulated according to the predicted deposition amount. The amount of droplet deposition can meet the prescription requirement. The results show that the training variance of the ANN is 0.003, and thus, the model is stable and reliable. The outdoor tests show that the error between the predicted droplet deposition and actual droplet deposition is less than 20%. The ratio of droplet deposition to prescription value in each unit is approximately equal, and a variable spray operation under different conditions is realized.

## 1. Introduction

Crop diseases and weeds are important factors that affect crop yield and quality, and are mainly controlled through chemical pesticides. The plant-protection flight operation has also been changed from traditional artificial spraying to mechanical spraying [1]. Since the 1920s, the manned aircrafts have been used for agricultural production in the United States, which created a history of agricultural aviation [2]. The agricultural application of UAVs as a new application in the field of agricultural plant protection has been widely researched and applied [3,4]. In 2014, China’s “Central Document No. 1” proposed to promote the development of eco-friendly agriculture, and especially pointed out that the construction of agricultural aviation should be strengthened. In order to implement document No. 1 of the Central Committee, the Ministry of Agriculture formulated the Action Plan for Zero Increase in Pesticide Use by 2020 [5]. It proposed that we should vigorously promote pesticide reduction and control of pesticides, actively explore the way to develop modern agriculture with high efficiency, product safety, resource saving and environmental friendliness, and strive to achieve zero increase in total pesticide use by 2020. Pesticides application by UAV can reduce the wastage of pesticides to a certain extent. However, owing the influence of environmental factors and rotor wind field, UAVs suffer from droplet drift and droplet sticking [6]. The main pesticide application methods currently used in China is unified spraying [7], and the utilization rate of pesticides is low. It is estimated that approximately 2.5 million thousand tons of insecticides are used globally every year [8]. According to statistics, the utilization rate of pesticides in China by 2017 was only 20% [9]. Variable spray technology can be applied to spray objects on demand, and the potential of variable spray in improving pesticide utilization, reducing pesticide residues and reducing environmental risk has been formed under the international consensus [10]. 

Recently, variable spray technology has been extensively studied. Cruvinel et al. [11] built relations among running speed, nozzles height, and spray flow. This method only regulates the flow rate according to speed and height, to achieve the purpose of uniform spraying. However, environmental factors and spray system parameters influence the spray effect [12]. Ambient temperature, humidity and wind speed cause droplet drift and evaporation, which affect droplet deposition. The pitch between nozzles in the structural parameters of the fuselage has a certain effect on the uniformity of spray [13]. Moreover, the pitch of the rotor changes its wind field and affects the droplet deposition [14]. Deng et al [15] built a constant-pressure sprayer that was controlled by a closed-loop proportional integral derivative algorithm. In this sprayer, the pressure was changed by adjusting the opening of the solenoid valve, thus changing the spray volume. In the same field, crops suffer from different severities of pests and diseases, and environmental factors and flight parameters influence the spray effect. Wang et al. [16] set different wind speeds and flight angles to simulate the spray operation of UAV in a wind tunnel. Finally, a relation model between the influence factors and spray volume was built. However, the traditional linear relation model of traffic flow and various factors is not flexible enough, it is impossible to distinguish the influence degree of each factor. Because environmental parameters and flight parameters change in real time during plant protection operations, the linear relation model between spraying amount and flight speed at the flight altitude cannot accurately adjust the flow rate [17]. It is necessary to establish a dynamic model between influencing factors and spraying amount. 

An artificial neural network (ANN) is a large-scale parallel nonlinear dynamic system. And neural network is a mathematical model used to find the relation between input and output datasets with complex relations [18,19]. With its successful application in various fields, ANN technology has entered the agricultural field and been successfully applied to various agricultural production problems. Through continuous monitoring, measurement and analysis of various physical phenomena, the development of complex agricultural ecosystems can be better understood and predicted [20]. Recently, the application of ANN technology in plant protection spraying has been extensively studied. Patel et al. [21] studied the relations among temperature, flow rate, solvent atomization parameters and droplet size. An ANN model of input and output was established and its validity was verified experimentally. Chen et al. [22] established the error back propagation (BP) neural network model of water quality and its influencing factors to predict water quality. Amir Azizpanah et al. [23] combined the ANN with image processing technology to establish a spray drift prediction model under different conditions. The inputs of the neural network model include height, flow rate, pressure and wind speed. The outputs include droplet volume and diameter. This study demonstrates the important advantage of a neural network in predicting drift, but it is not combined with the actual plant protection operation. 

In this study, the factors influencing the spray effect during the operation of plant protection UAVs are comprehensively considered, which include flight parameters (altitude and velocity), environmental parameters (temperature, humidity and wind speed), and aircraft structural parameters (propeller pitch and nozzle pitch). The neural network model between the influence factors and droplet deposition is trained using considerable existing experimental data, originating from the published literature and our own experiments. The technology of plant integration is used to build a new intelligent variable spraying system for plant protection UAVs to improve the efficiency of agricultural aviation spraying, reduce the use of chemical pesticides, and increase crop yields.

The study is organized as follows: Section 2 introduces the design of neural network model. Section 3 introduces design of the plant protection UAV variable spray system based on neural network decision. Section 4 presents the performance evaluation of neural network model and the analysis of experimental results. Finally, Section 5 provides the concluding remarks.

## 2. Design of the Neural Network Model

### 2.1. Introduction of BP Neural Network

An ANN is an algorithmic mathematical model for distributed parallel information processing based on the behavioral characteristics [24]. Depending on the complexity of the system, this network can process information by adjusting the interconnection between several internal node. An ANN has the ability of self-adaptation and self-learning, and is more accurate than the conventional linear relation model [25].

**Figure 1:**
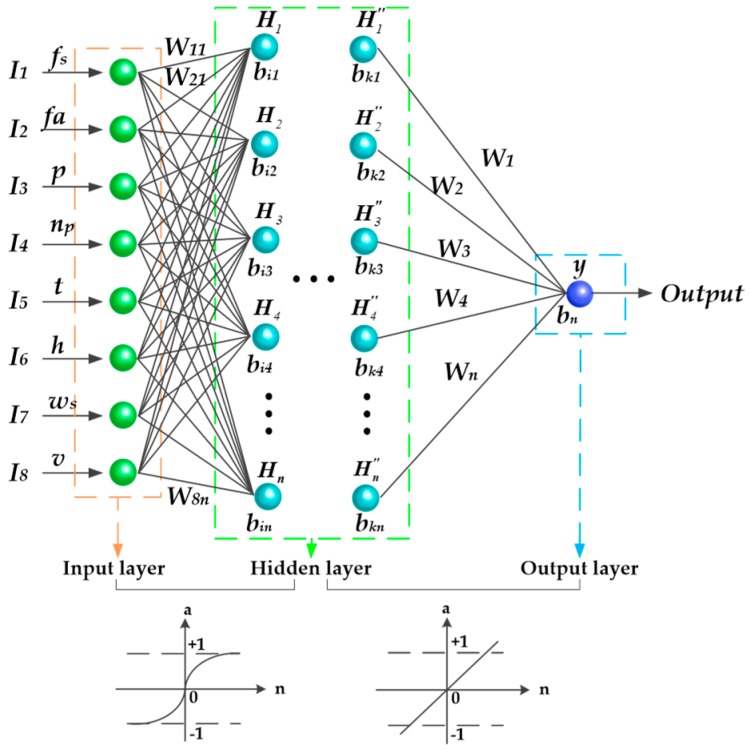
The topological structure of BP neural network. Note: *I_1_* ~ *I_8_* are input layer neurons; *H_1_* ~ *H_n_* and *H″_1_* ~ *H″_n_* are hidden layer neurons; *n* is the number of the hidden layer neurons. *y* is output layer neuron. *W_kn_* represents the weight between the *k*-th node of the input layer and the *n*-th node of the hidden layer. *b_in_* represents the bias between the *i*-th node of the input layer and the *n*-th node of the hidden layer. *b_kn_* represents the bias between the *k*-th node of the hidden layer and the *n*-th node of the hidden layer. *W_n_* represents the weight between the *n*-th node of the hidden layer and the output layer. *b_n_* represents the bias between the *n*-th node of the hidden layer and the output layer.

The core of an ANN for realizing its function is the algorithm [26]. The structure of neural network used in this study was the error BP neural network. The trained BP neural network could process the input information of similar samples by itself, and transformed it nonlinearly with the minimum output error. The topological structure of the BP neural network is shown in Figure 1.

The BP neural network model used in this study was a typical three-layer ANN model, which is widely used in non-linear modeling and solving interpolation problems such as adaptive, non-linear mapping and generalization abilities [27]. The basic concept was derived from that of the gradient descent method. The gradient search technique was used to minimize the error mean variance of the actual and expected output values of the network [28]. The neural network was divided into input layer, hidden layer and output layer. The neural network includes one or more hidden layers between the input layer and output layer, and it included one or more hidden layers between the input and output layers. In this study, the number of hidden layers was set to three and the input layer contained eight neurons. The flight speed (*f_s_*), flight altitude (*f_a_*), propeller pitch (*p*), nozzle pitch (*n_p_*), temperature (t), humidity (*h*), wind speed (*w_s_*) and prescription value (*v*) were taken as input, while the droplet deposition was taken as the output. Complete interconnection was adopted between two layers. 

A BP neural network is neural network with a learning function, which aims to obtain the relation between the input and output of the neural network from the training samples [29]. There is no mutual connection between the same layers. The neural network training comprises two steps: signal positive spread and error BP [30]. The sample data are transferred from the input layer to the hidden layer and processed to the output layer. The error is obtained by comparing with the actual value. If the actual output satisfies the error range, the output result will be obtained. If the actual output is not satisfied, the error will be transferred to the input layer through the hidden layer. In the next transmission process, the weight and bias of all input neurons will be adjusted until the error is reduced to a reasonable threshold [31], and finally, the training process is over. The flow diagram of the BP algorithm used in this study is shown in Table 1.

### 2.2. Database

#### 2.2.1. Sample Data Collection Experiment Scheme

**Figure 2:**
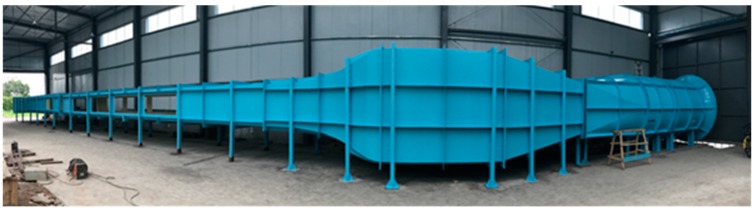
Wind tunnel laboratory.

BP Neural network training requires considerable sample data. The flight speed, flight altitude, propeller pitch, nozzle pitch, temperature, humidity, wind speed and prescription value are taken as input, while the droplet deposition is taken as the output. To improve the accuracy of neural network training, there are two main methods for obtaining the training sample data. One is to collect the relevant experimental data of multi-rotor UAVs by consulting the literature, and the other is to conduct the experiment through the wind tunnel laboratory of the National Center for International Collaboration Research on Precision Agricultural Aviation Pesticide Spraying Technology. The wind tunnel laboratory is shown in Figure 2.

**Figure 3:**
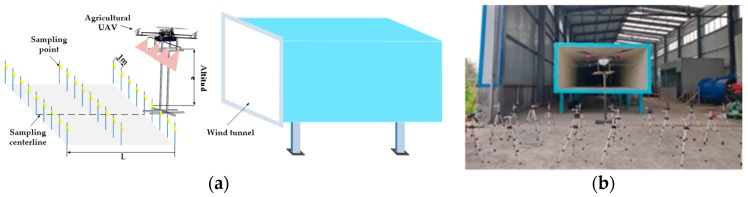
Schematic diagram of sample data collection experiment: (**a**) Sketch map of sample data collection; (**b**) Sample Data Acquisition Site; Note: L is the length of the sampling area.

Sample data collection in the wind tunnel environment was an important source of data in this study. The experiment was conducted in the wind tunnel laboratory of South China Agricultural University. The wind tunnel is a high/low-speed composite wind tunnel specially used for researching the agricultural aviation application technology. Because of the high probability of occurrence of diseases and insect pests at the tillering stage, the rice at tillering stage was simulated by 40–50 cm height brackets in the wind tunnel tests [32,33]. The experimental data collection covered the whole tillering stage to control various diseases and insect pests. According to the principles of accuracy, representativeness and statistics, the training samples used in this study were expected to cover most of the possibilities. That is, they should occur during the whole period of rice tillering stage for disease and pest control [34]. Therefore, the data collection experiment continued from June 2018 to December 2018. The simulated field experiments were conducted to collect relevant data. A schematic of the experiment scheme is shown in Figure 3a. The UAV was built of aluminum profiles (ES 3030, Yinjun Aluminum Co., Ltd., Guangdong, China). Along with the medicine box, a miniature diaphragm pump (PLD-2201, Shijiazhuang Prandi Co, Ltd., Shijiazhuang, China) and four pressure nozzles (110-015 types, LECHLER Company, Albstadt, Germany) were utilized. 

The pressure of nozzles was 0.3 MPa and the spray angle was 110°. Depending on the number of multi-rotor UAV rotors in China’s agricultural UAV market, 4, 6, 8 carbon fiber rotors were set up in the sample collection tests. UAV-relative blades were adjustable. According to the common multi-rotor UAV on the market, the adjustment range was 1.2–1.4 m. The distance between nozzles ranges from 0.45 to 0.55 m. The ranges of propeller pitch and nozzle spacing of UAVs included the parameters of all types of plant protection UAVs on the market. The sample data acquisition site is shown in Figure 3b.

#### 2.2.2. Sample Data Processing

**Figure 4:**
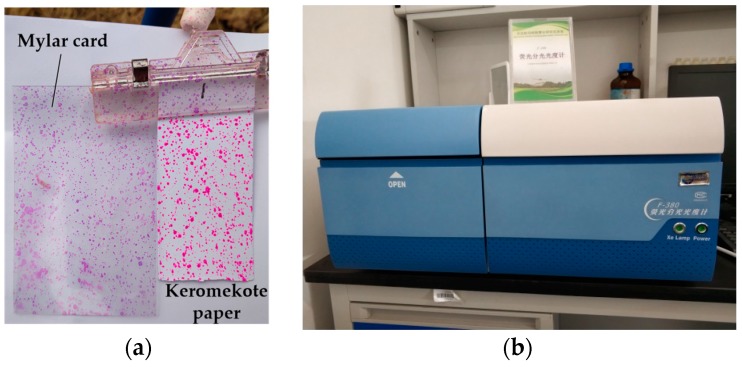
Data processing: (**a**) The collection of keromekote paper and Mylar card; (**b**) The fluorescence spectrophotometer.

A Rhodamine B (soluble fluorescent tracer) solution with 5 g/L concentration was used instead of the liquid to spray. Simultaneously, keromekote papers (30 mm × 70 mm) and Mylar cards (50 mm × 80 mm) were placed at each sampling point, which was located 50 cm away from the ground, to receive the deposited droplets. Three sampling bands with 1 m spacing were set up and nine sampling points with 1 m spacing were distributed in each sampling band. Control of rice diseases and insect pests at the tillering stage was simulated. After each test, the keromekote paper and Mylar card at each sampling point were collected, and then, brought back to the laboratory for analysis and treatment. The collection of the keromekote papers and Mylar cards during the experiment is shown in Figure 4a. 

**Figure 5:**
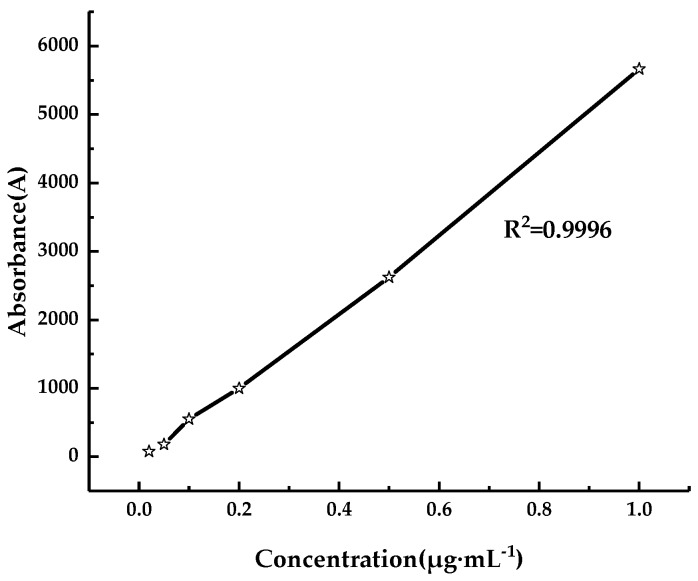
Standard curve between fluorescence and solution concentration.

The DepositScan software (USDA-ARS Application Technology Research Unit, Wooster, OH, USA) [35] was used to analyze the collected keromekote papers to obtain the droplet deposition per unit area of each sampling point [36]. The collected Mylar cards were eluted with 20 mL distilled water, then the eluted solution was placed in a fluorescence spectrophotometer (F380, Tianjing Gangdong Technology Development Co., Ltd., Tianjing, China) for analysis, as shown in Figure 4a. The fluorescence value of the standard solution was measured by configuring the standard solution with five gradients of 0.02, 0.05, 0.1, 0.2, 0.5 and 1 μg/mL. The labeling curve between the fluorescence and concentration was obtained by fitting, as shown in Figure 5.

Figure 5 shows the fitting relation between the fluorescence value and concentration. The linear fitting variance was 0.9996, and the fluorescence value of the eluted solution was obtained by elution. The concentration of Rhodamine B in the eluted solution was obtained according to the standard curve. Finally, the droplet deposition per unit area was obtained using the concentration. The formula is as follows [37]:

(1) $$\beta_{\mathrm{dep}}=\frac{(\rho_{sampl}-\rho_{blk})F_{cal}V_{dil}}{\rho_{spray}A_{col}}$$

where *β_dep_* is the amount of droplet deposition in g·cm^−2^; *ρ_sampl_* is the reading of the sample solution fluorescence meter; *ρ_blk_* is the reading of the fluorescence meter of the eluent; *F_cal_* is the calibration coefficient in g·L^−1^; *V_dil_* is the volume of the solution used to elute the collected sample in L; *ρ_spray_* is the concentration of the fluorescent tracer in the spray solution in %; *A_col_* is the area of the collected card in cm^2^.

#### 2.2.3. Sample Data Results

By analyzing the data obtained, the maximum, minimum, mean, standard deviation, skewness and kurtosis values of all sample data were counted. The statistical properties of the sample data are shown in Table 2. According to the data in the table, the flight speed and altitude in the sample data were within the range of flight parameters of plant protection UAVs. The ranges of temperature and humidity satisfied the conventional variation in rice temperature at the tillering stage in the Guangdong Province. The range of wind speed was 0.01 to 3.21 m/s. The wind speed was within a reasonable range of operational requirements of plant protection UAVs. The sample data were real and effective.

### 2.3. The Training Process of BP Neural Network

The BP neural network can learn and solve complex non-linear relations through experience. A different learning rate significantly influences the performance of a BP neural network model [38]. The smaller the learning rate, the slower is the convergence rate. If the learning rate is too high, to the model can easily oscillate. To reduce the training time and times of searching for the optimal learning rate, an adaptive learning rate is adopted. Thus the selection range is generally within [0.01, 0.8] [39]. The number of input layer nodes is *i*, and that of the output layer nodes is *k*. The number of hidden layers is *h*, which can be calculated by adopting the following empirical formula [40]:

(2) $$h=\sqrt{i+k}+t$$

In our neural network model constructed in this paper, the number of input layer nodes was eight, the number of output layer nodes was one. Thus, the number of hidden layers was three. The three layers of neurons were recorded as *H_j_*, *H′_j_* and *H″_j_* in turn. To improve the generalization ability of the network in the training process, the input data were normalized before the sample data were input to the neural network for training. The normalization formula is as follow [41]:

(3) $$X=(0.98-0.02)(\frac{X_{0}-X_{\min}}{X_{\max}-X_{\min}})+0.02$$

where *X* is the normalized parameter; *X_max_* is the maximum value of input data; *X_min_* is the minimum value of the input data; *X*_0_ is the original experimental data of the input layer.

**Figure 6:**
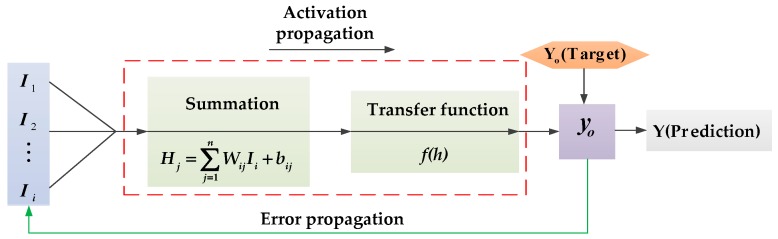
The basic neuron in the hidden layers in the feedback phase.

The input of the hidden layer neurons is the sum of weighted and biased vectors of the input neurons. Figure 6 shows the flow of a basic neuron in the hidden layer during the feedback phase. Then, the output signal of the neurons was generated by adding the non-linear transfer functions. The mathematical function of the process is described as [42]:

(4) $$y=f(\sum_{i=1}^{k}W_{ij}I_{i}+b_{ij})$$

where *W_ij_* is the weight that connects the input neuron of the previous layer to the neuron of the current layer; *I_i_* is the input component; *b_ij_* is the bias associated with the neuron of the current layer; *f(t)* is the logarithmic Sigmoid type function between the input layer and the first hidden layer.

Then, the output values of the data were obtained after the hidden layer operation. The formula for calculating the output value is as follow [43]:

(5) $$y=g(\sum_{j=1}^{n}W_{j}H^{\prime \prime }{}_{j}+b_{j})$$

where *W_j_* is the weight that connects the neuron of the last hidden layer to the neuron of the output layer; *H″_j_* is the component of the last hidden layer; *b_j_* is the bias associated with the neuron of the output layer; *g(t)* is the linear function between the last hidden layer and the output layer.

The predicted value was obtained by neural network operation. The error was calculated through a comparison of the predicted value with the actual value. The formula is as follow [44]:

(6) $$E=\frac{1}{2}{\sum_{j=1}^{n}\left(y-y_{0}\right)}^{2}=\frac{1}{2}\sum_{j=1}^{n}e_{j}^{2}$$

where *y* is the value of the hidden layer output; *y_0_* is the actual value or expected output value.

A reasonable error range was set. If *E* is within the error range, the network training will end, otherwise, the error BP process will take place and the weight and bias of each layer are update. The weight and bias updating formula from the hidden layer to the output layer are as follows [45]:

(7) $$W_{ij}=W_{ij}+\alpha H_{j}e_{j}$$

(8) $$b_{j}=b_{j}+\beta e_{j}$$

The weight and bias updating formula from the input layer to the hidden layer are as follows [46]:

(9) $$W_{ij}=W_{ij}+\eta H_{j}(1-H_{j})x_{i}\sum_{i=1}^{k}W_{ij}e_{j}$$

(10) $$b_{j}=b_{j}+\lambda H_{j}(1-H_{j})x_{i}\sum_{j=1}^{k}W_{ij}e_{j}$$

where *α*, *β*, *λ* and *η* are the coefficients. After the *E* calculated by Equation (6) is within the error range, the predicted value output from the output layer. 

### 2.4. Performances of BP Neural Network Models

To evaluate the stability and sensitivity of the ANN model, four statistical parameters were adopted, namely the coefficient of correlation (*r*), the root mean square error (*RMSE*), the overall index of model performance (*OI*), and the mean absolute error (*MAE*) [47]. The formulas for calculating the indices are defined as follows:

(11) $$r=\frac{\sum_{i=1}^{m}\left(X_{i}-\bar{X})(Y_{i}-\bar{Y}\right)}{\sqrt{\sum_{i=1}^{m}{\left(X_{i}-\bar{X}\right)}^{2}{\sum_{i=1}^{m}\left(Y_{i}-\bar{Y}\right)}^{2}}}$$

(12) $$RMSE=\sqrt{\frac{\sum_{i=1}^{m}{\left(X_{i}-Y_{i}\right)}^{2}}{m}}$$

(13) $$OI=\frac{1}{2}\left(2-\frac{RMSE}{X_{\max}-X_{\min}}+\frac{\sum_{i=1}^{m}{\left(X_{i}-Y_{i}\right)}^{2}}{\sum_{i=1}^{m}{\left(X_{i}-\bar{X}\right)}^{2}}\right)$$

(14) $$MAE=\frac{\sum_{i=1}^{m}\left|X_{i}-Y_{i}\right|}{m}$$

where *X_i_* is the training value; *Y_i_* is the predicted value; *m* is the training steps; $\bar{X}$ is the average training value; $\bar{Y}$ is the average predicted value; *X_max_* is the maximum training value; *X_min_* is the minimum training value;

The *r* is used to measure the correlation between the predicted and actual values. The closer the *r* value is to 1, the higher is the accuracy of the representation model. *RMSE* is used to describe the variance between the predicted value and actual values in the sample training process. *RMSE* characterizes the accuracy of the model. *OI* Represents the Fitting Accuracy of an ANN Model. The closer the *OI* value is to 1, the higher is the fitting accuracy of the model. The lower the *MAE*, the better the training effect.

The Kolmogorov theorem shows that the BP neural network can achieve an arbitrary non-linear function approximation, when the structure of the BP neural network satisfies the three-layer structure with the input layer, hidden side and output layer, and the number of neurons in the hidden layer is sufficient [48]. The performance of the BP neural network is related to the number of hidden neurons. In general, the more the hidden neurons, the better the network performance [49]. To determine the number of neurons in the hidden layer in this study, the relevant parameters of different numbers of neurons in the hidden layer were measured as shown in Table 3.

As shown in the table, when the number of neurons in the hidden layer is 20, the values of *r*, *OI* and *MAE* of the model reach the peak. When the numbers of hidden layer neurons were increased from 12 to 20, the values of *r*, *OI* and *MAE* increased by 14.69%, 1.93% and 17.99%, respectively. The *RMSE* value continued to decrease with an increase in the number of neurons in the hidden layer. When the number of neurons in the hidden layer exceeded 20, the values of *r*, *OI* and *MAE* decreased. Therefore, in this study, the optimal number of hidden layer neurons in the design of the neural network structure was 20.

**Figure 7:**
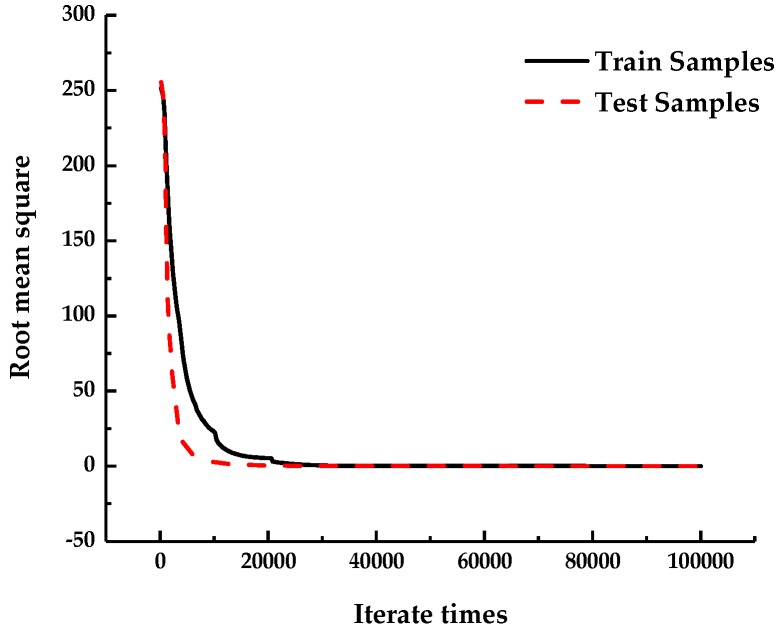
The RMSE of the network training.

Owing to the errors of the sample data, it is easy to over-fit in the training process of the neural network model. On the surface of this phenomenon, the sample training appears to fit well and cannot reflect the real mapping relation. After importing the sample data, 30% of the total sample data were selected as the test set automatically, and the remaining 70% of the total sample data were used as the training set. During training, two types of data were stored in different arrays. In each step of the training process, the test samples were used to evaluate the error of the current network. When the error of the test sample was increased gradually, the training was stopped. Figure 7 shows the *RMSE* of the network training.

As shown in Figure 7, in the training process of the sample data model, the error decreased rapidly with the number of iterations and converged quickly to below the set expected error. In the initial stage of training, the descending range of the training samples was similar to the test samples, indicating that the BP neural network fast-approached the real input-output mapping relation as a whole. The root mean squares of the training and test samples were stable in the set threshold range, indicating that the neural network model was stable and reliable.

**Figure 8:**
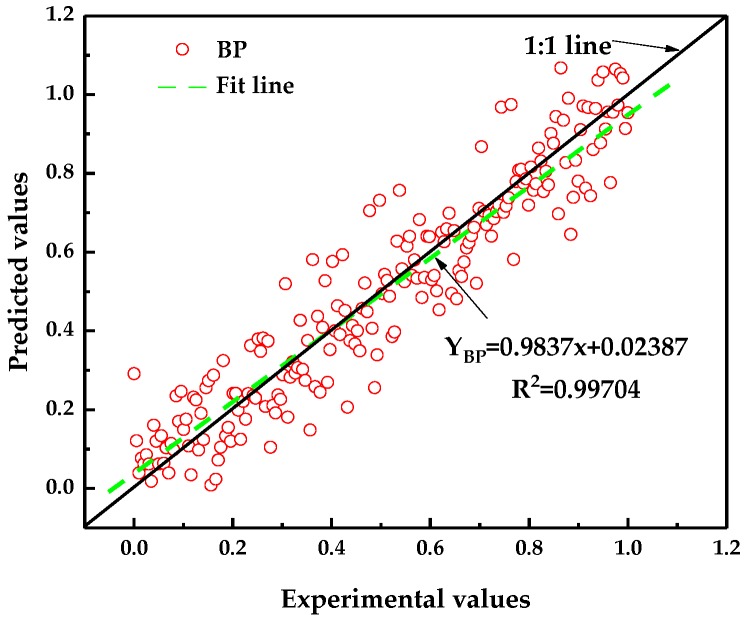
Comparison of training predicted value and experimental value of BP neural network.

Figure 8 shows a comparison between the predicted and experimental values during training. Under the condition where the prediction error was neglected, the ratio between the predicted and the experimental values was stable at approximately 1:1. The slope of the fitting line was 0.9837, which is approximately 1. The *R*^2^ of the fitting straight line was 0.99704. The results showed that the prediction by the BP neural network model can achieve high accuracy matching between the predicted and experimental data.

## 3. Design of Variable Spray System 

### 3.1. Working Principle of Variable Spray System

**Figure 9:**
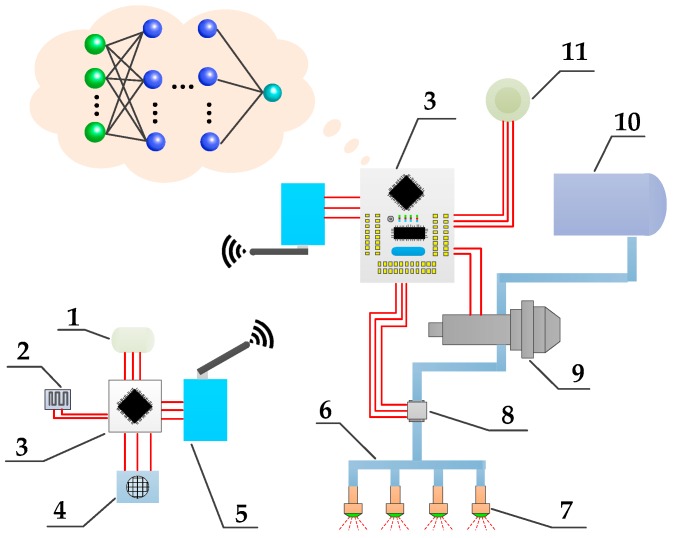
The structure of variable spray system based on BP neural network. Note: 1. Temperature sensor; 2. Humidity sensor; 3. STM32 control chip; 4. Wind speed sensor; 5. Wireless transmission station; 6. Compression pipe; 7. Pressure nozzle; 8. Flow sensor; 9. Micro-diaphragm pump; 10. Medicine box; 11. GPS.

In this study, a variable spray system including the variable rate spray system based on the BP neural network decision model and environmental meteorological collection module was established. A schematic of the plant protection UAV variable spray system designed by our research team is shown in Figure 9. 

First, a BP neural network model was constructed in the spraying decision control system and trained using the data. The majority of the data were obtained from our own experiments, while some was taken from the published literature. These data included all test data of multi-rotor UAVs on the market. The training results were stored in the decision module. Then, the environmental information collection module collected real-time environmental temperature, humidity and wind speed data, which were transmitted to the neural network decision module through a wireless transmission station. Simultaneously, the position and flight parameter information were acquired by the UAV airborne Global Position System (GPS). The operating prescription values were obtained according to the location information. Finally, the amount of deposition was obtained through the neural network operation and the flow rate of the variable spray system was adjusted according to the amount of deposition.

**Figure 10:**
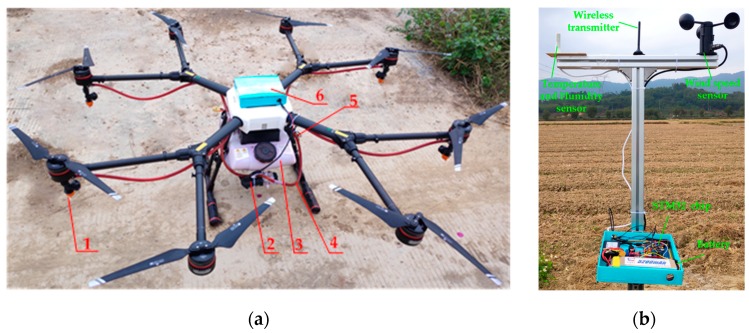
The physical drawings of the system. (**a**) Variable rate spray system based on BP neural network decision. (**b**) Environmental meteorological collection module. Note: (1) Pressure nozzle. (2) Micro-diaphragm pump. (3) Medicine box. (4) Compression pipe. (5) Wireless transmission station. (6) STM32 controller.

The physical drawings of the neural network decision module and variable spray actuator module are shown in Figure 10. The STMicroelectronics-32 (STM32) chip (Yusong Electronic Technology Co., Ltd. Shenzhen, China) was chosen as the control core. The variable rate spray system based on the BP neural network decision model was installed in the UAV (MG-1, DJ-Innovation Technology Co., Ltd., Shenzhen, China), as shown in Figure 10a, along with the medicine box, a miniature diaphragm pump (PLD-2201, Shijiazhuang Prandi Co, Ltd., Shijiazhuang, China). The wireless transmission stations (Heideweiye Technology Co., Ltd., Shenzhen, China) were used to transmit the data. The environmental meteorological collection module was mainly used to measure the environment information, as shown in Figure 10b. The temperature and humidity sensors (Miaoguan Technology Co., Ltd., Zhejiang, China) were used to measure real-time temperature and humidity. A high-precision wind speed sensor (Huakong Technology Co., Ltd., Beijing, China) was used to measure the wind speed data. The measurement accuracy was 0.01 m/s.

The deposit volume was predicted by the local neural network decision module, and the flow rate of the system was obtained according to the jet amplitude and flight speed of the UAV. The square wave of pulse width modulation (PWM) was generated by the STM32 controller to adjust the micro-diaphragm pump in order to regulate the system flow rate. Among them, the relation between the flow rate of the micro-diaphragm pump and the duty ratio of the PWM square wave was fitted using a cubic polynomial. The relational can be expressed as [50]:

(15) $$D=(20.776v^{3}-21.452v^{2}+8.242v-0.431)\times 100\%$$

where *v* is the flow rate of single nozzle in L/min, and *D* is the duty ratio of PWM square wave in %.

### 3.2. Design of the System Program

**Figure 11:**
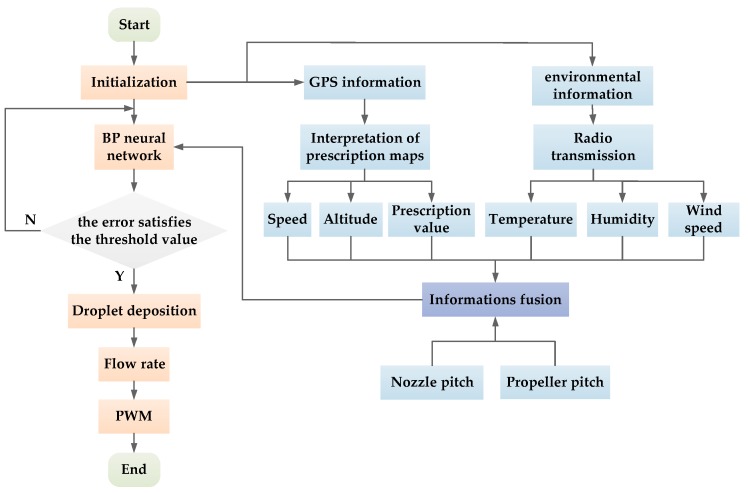
Program flow chart.

The system program was written in Keil Software (ARM Germany GmbH, Texas, USA) with C language. The system program included a BP neural network model, sensor data collection, prescription value acquisition and PWM square wave generation. The program flowchart is shown in Figure 11.

When the system operated, the flight parameters (speed and altitude) and position information of the UAV were obtained using the communication protocol (NMEA0183) of GPS. The spraying operation prescription value of the UAV’s current flying position was determined by location information matching. The prescription value of the current position was obtained by matching the position of the UAV with that of the prescription map [51]. The sensors acquired temperature, humidity and wind speed data, which were transmitted to airborne controllers in real time through wireless transmission stations. After information fusion, the information was used as input to the BP neural network to predict the deposition. The flow rate of the spraying system was obtained by combining the flight speed of the UAV with the predicted deposition amount. Finally, a PWM square wave was generated to complete the flow rate regulation process.

## 4. Experiments

### 4.1. Experiment Scheme

**Figure 12:**
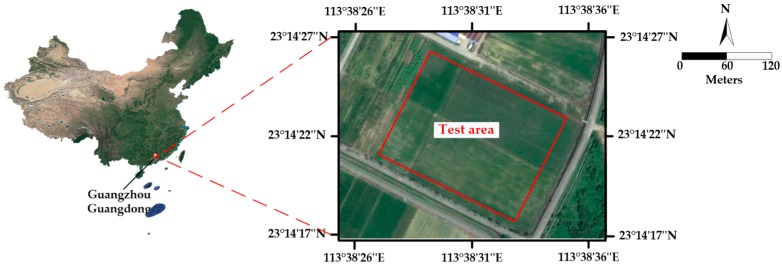
Location of the experimental site.

To verify that the system can change the spraying flow rate with environmental factors, flight parameters, severity of pests and diseases and UAV structural parameters. The outdoor spraying experiments were conducted using the designed system. A 60 m × 80 m field was selected as the experimental site located in a paddy field of Zengcheng Scientific Research and Teaching Base of South China Agricultural University, Guangzhou, China. The location of the experimental site is shown in Figure 12. 

**Figure 13:**
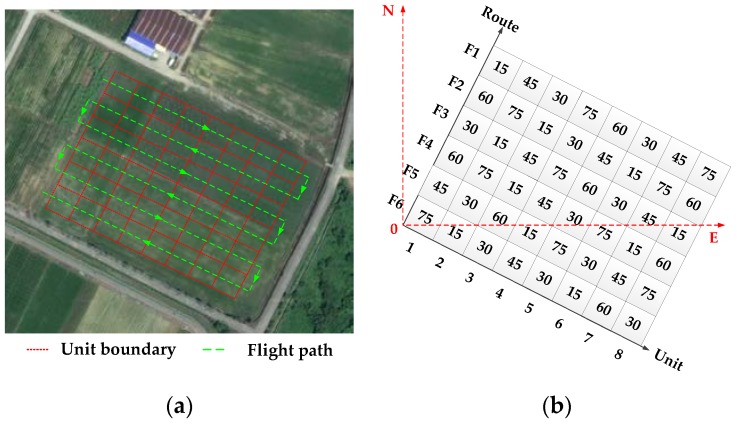
(**a**) Operating unit; (**b**) Prescription value of each unit.

The experimental site was divided into 10 m × 10 m units. To collect data conveniently, the route of the UAV spraying operation was planned. The unit division of the operation plot and route planning of the UAV spraying operation are shown in Figure 13a. Combined with the guidance of the pest control expert system, the spraying prescription values of each unit in the working plot were set by the linear interpolation method. The following five gradient levels of dosage were set up: 15, 30, 45, 60, and 75 L/hm^2^. The spraying prescription values of each unit were wet as shown in Figure 13b.

**Figure 14:**
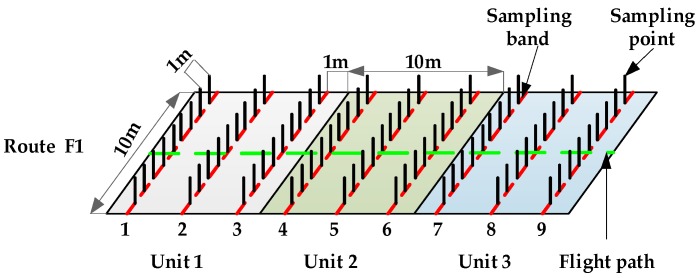
The diagram of the sampling bands.

As seen from Figure 13, the test had the following six routes, F1−F6, and each unit had eight operation units. Droplet deposition and deposition density per unit area are important parameters for reflecting the spray effect [52]. To verify that under different prescription values, the designed system can comprehensively consider environment, flight and structural parameters to make the deposition satisfy requirements of the prescription value, sampling bands were set in each operation unit as shown in Figure 14. 

The figure shows only the layout of sampling points in unit1-unit3 on route F1. The sampling band arrangement of the operation units on the other three routes was the same as route F1. Sampling bands such as 2, 5, and 8 were set at the center line of each operation unit. Because new prescription values were obtained when UAV passes through the demarcation line of the operation unit, a sampling band was set at the position of 1 m from the demarcation line on both sides of the operation unit demarcation line. There were nine sampling points in each sampling band, namely −4#, −3#, −2#, −1#, 0#, 1#, 2#, 3#, and 4#. The sampling devices were installed at each sampling point to collect droplet deposition.

### 4.2. Experiment Data Acquisition

**Figure 15:**
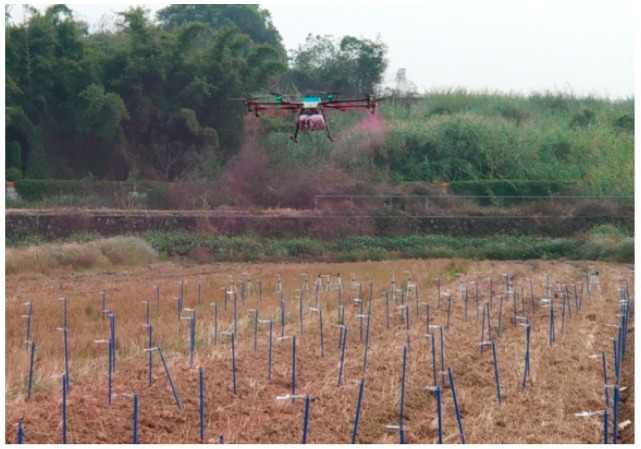
The spray test site.

The outdoor experiment was conducted from 10:00 to 13:00 on January 8, 2019. The average temperature and ambient humidity were 21.6 °C and 70%, respectively. The wind speed was stable below 3.0 m/s. The ambient wind direction is always northeasterly (approximately perpendicular to the flight route). Four repeated experiments were conducted. The variable spray control system based on the BP neural network decision was performed using eight rotor plant protection UAV (MG-1, DJ-Innovation Technology Co., Ltd., Shenzhen, China). During the experiment, the environmental parameter measurement sensors were fixed 2 m away from the ground by the field. The test site is shown in Figure 15.

The DepositScan software and fluorescence spectrophotometer were used to analyze the keromekote papers and Mylar cards. The data processing method is the same as that shown in Section 2.2.2. The droplet depositions were counted.

### 4.3. Experiment Results

#### 4.3.1. Predicted and Experimental Depositions

**Figure 16:**
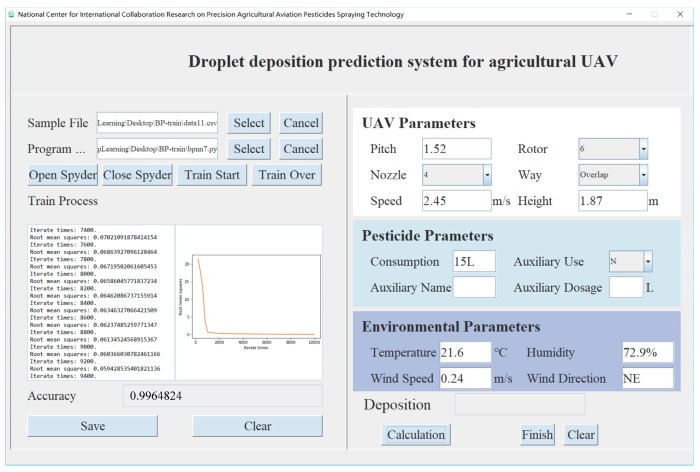
Software interface for droplet deposition prediction system for agricultural UAV.

Based on the training of the BP neural network model, the software named Droplet Deposition Prediction System for agricultural UAV (UAVDDPS) was designed and developed to predict droplet deposition for agricultural UAVs. The software interface is shown in Figure 16.

**Figure 17:**
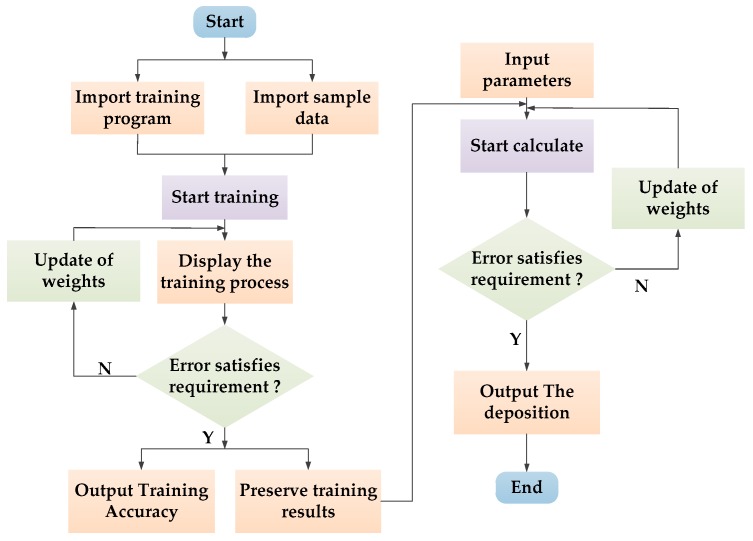
The software workflow diagram.

The working principle of the software is divided into two parts: model training and data prediction. The software workflow diagram is shown in Figure 17. The model was trained by the collected sample data and the BP neural network program. After the training, the results were saved. Then, the UAV system, pesticide and environmental parameters were input manually. The UAV system parameters included the pitch, rotor, nozzle, pattern, speed and height. The pesticide parameters included the consumption, auxiliary use, auxiliary name and the auxiliary dosage. The environmental parameters included the temperature, humidity, wind speed and wind direction. The saved training model was used for the calculation. When the error did not satisfy the set threshold requirement, the error propagated backward and the weight was updated until the error satisfied the requirement. Finally, the deposition was calculated using the BP neural network model.

The plant protection UAV selected in the test was a eight-rotor UAV with a relative spacing of 1.52 m between the two rotors. Four pressure nozzles were mounted below four opposite rotors. The environmental information and flight parameter information received by each sensor during the experiment were stored in an SD card. After the experiment was completed, the relevant information of each sampling band was obtained by analyzing the stored data. The droplet depositions were predicted by the UAVDDPS software. The droplet depositions at the center line of each unit on route F1 in the first test and route F2 in the fourth test were selected for analysis. The comparison results of the predicted deposition and elution deposition are shown in Table 4. 

From the table, the errors between the droplet deposits collected by the experiment and those predicted by the BP neural network are within 20%. The droplet deposition amount of the actual operation can satisfy the prescription value requirements. The prescription values of the operation units corresponding to acquisition bands F1-1–F1-23 are as follows: 15, 45, 30, 75, 60, 30, 45, and 75 L/hm^2^. The normalized ratio of droplet deposition predicted by the BP neural network is 1:3.69:2.06:4.95:3.99:2.01:3.098:5.02. The normalized ratio of droplet deposition found experimentally is 1:3.07:1.95:4.41:3.97:2.01:2.64:5.85. The prescription values of the operation units corresponding to the acquisition bands F2-1–F2-23 are as follows: 60, 75, 15, 30, 45, 15, 75, and 60 L/hm^2^. The normalized ratio of droplet deposition predicted by the BP neural network is 3.71:4.27:0.79:1.66:2.38:1:4.37:4.15. The normalized ratio of droplet deposition found experimentally is 4.04:4.72:1.04:2.12:2.80:1:5.91:4.18. The ratio of droplet deposition at the center line of each unit to the prescription value is approximately equal. The small difference is mainly due to the drift of droplets caused by the rotor wind field during operation. The experimental results show that using experimental data to train the neural network model between droplet deposition and its influencing factors is stable and reliable. The designed variable spray decision system can predict the droplet deposition quickly according to the parameters of the sensor feedback and the prescription value of the operation plots. The system can accurately regulate the flow according to the predicted deposition amount, so that the droplet deposition amount after operation is satisfied with the prescription value requirement. 

#### 4.3.2. Droplet Deposition Analysis

To verify the function of the variable spray system, four repetitive tests were conducted in outdoor experiments, whose route and prescription values are the same as those shown in Figure 13. 

**Figure 18:**
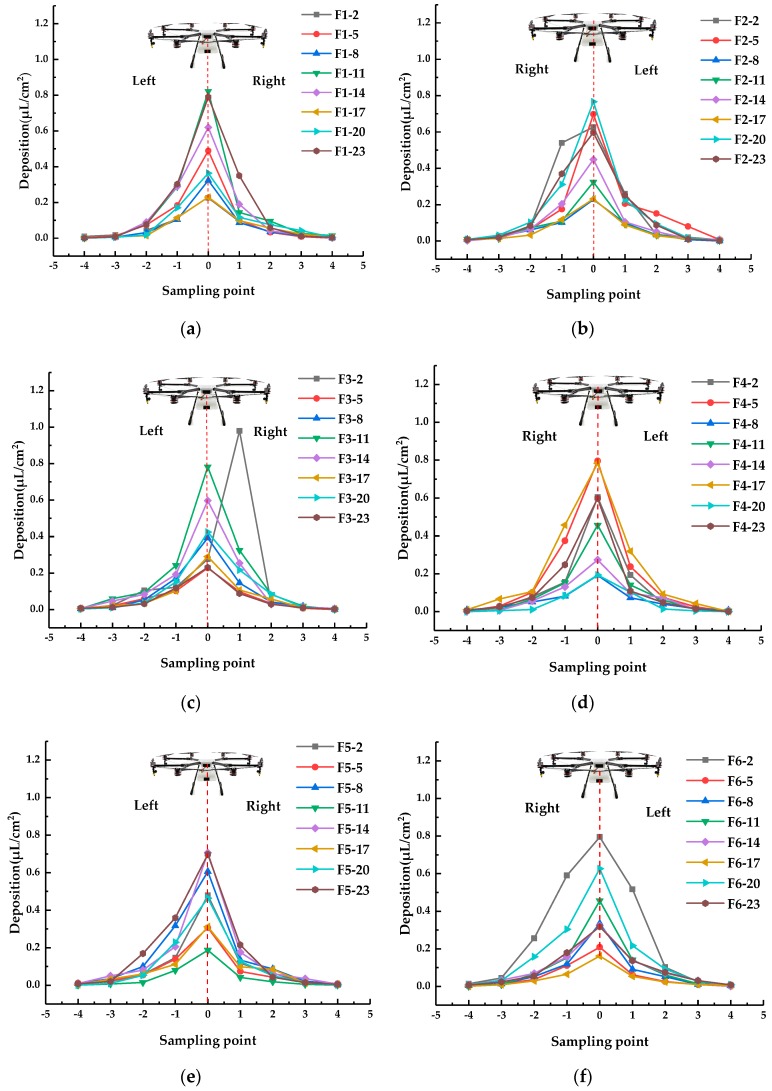
Droplet deposition at different sampling points at the center line of operation unit. (**a**) Route F1. (**b**) Route F2. (**c**) Route F3. (**d**) Route F4. (**e**) Route F5. (**f**) Route F6.

Droplets were collected by the keromekote papers set at each sampling point. The droplet deposition in each operation unit of the third test was selected for the analysis. The number of droplets collected is shown in Figure 18. 

As shown in the figure, the deposition trend of the droplets is the same in different operation units, i.e., normal distribution. The deposition amount of each unit is related to the spraying prescription value. The peak value of deposition on the sampling band is near the sampling center line, mainly because the droplets at both ends of the operation are drifted and rolled up by the rotor wind field. The droplets below the fuselage settle under the action of the downward-pressure wind field of the rotor. The wind speed was less than 3 m/s, and the peak value of droplet deposition on each sampling line was 0#. The peak value of sampling band F3-2 in Figure 18c is found to be at sampling point 1#, through the analysis of environmental factors and flight trajectories. The UAV body deviates from the sampling center line while entering route F3 from route F2. The wind speed in the natural environment has some effect on spray deposition during the experiment. For example, the droplet deposition on the right side of the fuselage is more than that on the left side, as shown in Figure 18b. During the experiment, the amount of droplet deposition is mainly distributed in the effective sampling points of −3#, −2#, −1#, 0#, 1#, and 2#. The effective spraying swath meets the basic requirements of aviation low-capacity plant protection spray [53].

#### 4.3.3. Deposition of the Boundary of Operation Unit

**Figure 19:**
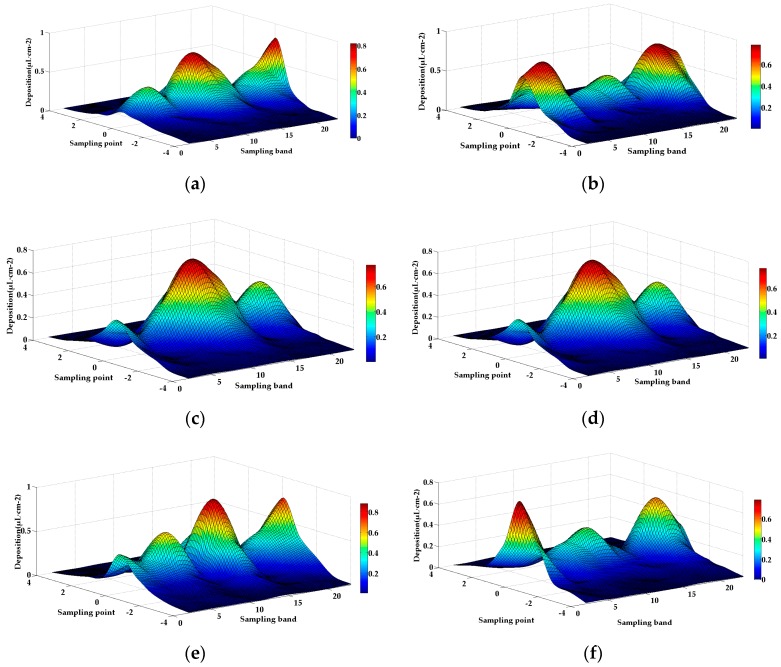
Fourth test of the droplet deposition amount of operating units on different routes. (**a**) Route F1. (**b**) Route F2. (**c**) Route F3. (**d**) Route F4. (**e**) Route F5. (**f**) Route F6.

The UAV passes through the boundary line of the operation unit, new prescription values are obtained. To study the droplet deposition at the operation unit boundary, the experimental data of the fourth repeated experiment were analyzed. Table 5 shows the amount of droplet deposition in three units of operation route F1. Droplet deposition on the other routes is shown in Figure 19.

From Table 5, the droplet deposition at the center line of each unit is approximately equal to that at both sides of the center line. The deviation is stable below 10%. Different prescription values correspond to different droplet deposits in the unit. The ratio of deposits to prescription values is approximately equal, as shown in Figure 19. The sampling bands F1-4, F1-7, F1-10, F-13, F1-16, F1-19 and F1-22 are the sampling bands 1 m behind the boundary. The deviations between the droplet deposition on these sampling belts and that at the centerline of the current unit are 8.04%, 9.03%, 3.11%, 2.43%, 4.08%, 2.97%, and 2.1%, respectively.

The flow change of the plant protection UAV has been completed in the process of flying 1 m from the demarcation line. According to the operation speed of the plant protection UAV 2–4 m/s, the system takes less than 0.25 s from receiving a new flow to changing the flow to the target value, which reflects the sensitivity of the system. In Figure 19, the droplet deposition of each operation unit changes clearly. The droplet deposition distribution in the sampling band shows a normal distribution trend, which indicates that the system can accomplish variable spray operation according to the prescription value and influencing factors.

## 5. Conclusions

Variable spray technology can achieve the goal of applying pesticide on demand. While improving the chemical effects of pesticides, and effectively reducing the use of chemical pesticides. In this study, artificial neural network is used to study the variable spraying system of plant protection UAV:

(1) Based on the existing data of plant protection UAV operation, combined with the error back propagation neural network technology, a neural network model which affects the spray droplet deposition factor and deposition volume was trained. These factors include environment temperature, humidity, wind speed, flight speed, flight altitude, prescription value, nozzle pitch and propeller pitch. The training error of the BP neural network is 0.003.
(2) The variable spray technology is combined with BP neural network technology to predict spray deposition in real time. The droplet depositions meet the prescription value requirements. The error between the predicted droplet deposition and actual droplet deposition is less than 20%.
(3) The UAV variable spray system based on neural network is evenly sprayed. From the change of prescription value to the response time of regulated flow is within 0.25 s, the spray range meets the operational requirements of plant protection UAVs.

## Figures and Tables

**Table 1:** The code of the BP neural network algorithm.

**Algorithm 1:** The back propagation neural network
**Definition:** Input layer neurons *x_i_*; The number of input layer neurons *n*; The hidden layer neurons *H_j_*, *H′_j_* and *H″_j_*; The number of input layer neurons *k*; The output layer neurons *y* Deposition **Initialization:**
Initialize all weights and biases in network; **for** *i*=1 **to** *n* **do** Create hierarchy model and assign to neurons *x_8_=*(*f_s_*, *f_h_*, *p*, *n_s_*, *t*, *h*, *w_s_*, *v*); *y=*(*d*) *x_i_ = X*(*x_i_*) **end**
**for***j*=1 **to***k***do** **for***t*=1 **to** 3 **do** *H_j_*←*ΣW_ij_x_i_+b_j_* *H′_j_*←*ΣW^’^_ij_H_j_+b^’^_j_* *H″_j_*←*ΣW″_ij_H′_j_+b″_j_* *y_o_*←*g*(*H″_j_*) **end** **end** **for all** *j* **in** *k* **do** *E*←*1/2Σe_j_^2^*
**if** (*E* ∉ *Error*) **do** W_ij_←W_ij_+*αH_j_e_j_* *b_j_*←*b_j_+βe_j_* **return** *i=1*
**else** *y=y_o_*
**End**

**Table 2:** Statistical properties of sample data.

Parameters	Max	Min	Mean	Std.Dev	Skewness	Kurtosis
Flight speed (m/s)	5.22	1.00	3.010	0.9847	0.37	−0.33
Flight altitude (m)	4.08	1.45	1.920	0.691	0.83	0.91
Temperature (°C)	32.00	25.00	28.770	1.750	−0.92	−0.26
Humidity	0.741	0.45	0.643	0.083	−0.82	−0.72
Propeller pitch (m)	1.40	1.20	1.310	0.099	−0.20	−1.98
Nozzle pitch (m)	0.55	0.45	0.513	0.038	−0.47	−1.14
Wind speed (m/s)	3.20	0.01	1.144	0.585	0.76	0.88
Prescription (L/hm^2^)	48.00	5.00	20.50	9.548	0.12	−0.96
Deposition (μL/cm^2^)	8.86	0.01	5.69	15.72	4.36	20.27

**Table 3:** Statistical performance of BP neural network model with different numbers of hidden layer neurons.

Number of Hidden Layer Neurons	Statistical Parameters			
	*r*	*RMSE* (%)	*OI*	*MAE* (%)
12	0.864	4.673	0.933	3.008
14	0.907	4.651	0.936	3.270
16	0.953	4.643	0.942	3.445
18	0.980	4.321	0.946	3.507
20	0.991	4.215	0.951	3.549
22	0.976	4.185	0.949	3.498
24	0.952	4.137	0.945	3.452

**Table 4:** The comparison between predicted and experimental depositions.

Band	Temperature (°C)	Humidity	Speed (m·s^−1^)	Height (m)	Wind Speed (m·s^−1^)	Predicted Deposition (μL·cm^−2^)	Experimental Deposition (μL·cm^−2^)	Deviation (%)
F1-2	20.65	72.9%	2.07	1.94	0.25	0.3058	0.317	3.66
F1-5	20.65	72.9%	2.26	2.13	0.23	1.1284	0.974	13.68
F1-8	20.65	72.8%	2.45	2.15	0.17	0.6291	0.613	2.56
F1-11	20.65	72.8%	2.89	2.21	0.21	1.5137	1.396	7.78
F1-14	20.65	72.8%	2.25	2.17	0.23	1.2231	1.258	2.85
F1-17	20.65	72.8%	2.31	2.05	0.19	0.6145	0.636	3.50
F1-20	20.65	72.8%	2.46	2.12	0.21	0.9473	0.886	6.47
F1-23	20.65	72.8%	2.43	2.14	0.20	1.5348	1.756	14.41
F2-2	21.91	68.6%	3.16	2.06	1.08	1.3961	1.265	9.39
F2-5	21.91	68.6%	3.25	2.27	0.98	1.6087	1.476	8.25
F2-8	21.91	68.6%	3.30	2.24	0.67	0.2973	0.324	8.98
F2-11	21.91	68.6%	3.35	2.35	0.84	0.6239	0.662	6.11
F2-14	21.91	68.6%	3.41	1.94	1.05	0.8953	0.876	2.16
F2-17	21.91	68.6%	3.75	1.68	1.14	0.3769	0.313	16.95
F2-20	21.91	68.6%	3.70	1.75	1.32	1.6481	1.849	12.19
F2-23	21.91	68.6%	3.59	1.45	1.09	1.5649	1.307	16.48

**Table 5:** The amount of droplet deposition in three units of operation route F1.

Unit	Prescription Value (L·hm^−2^)	Band	Deposition (μL·cm^−2^)	Coefficient of Variation
1	15	F1-2(Centerline)	0.347	9.46%
		F1-3	0.317	
2	45	F1-4	0.821	8.04%
		F1-5(Centerline)	0.887	
				1.24%
		F1-6	0.876	
3	30	F1-7	0.742	9.03%
		F1-8(Centerline)	0.675	
				2.37%
		F1-9	0.691	
4	75	F1-10	1.705	3.11%
		F1-11(Centerline)	1.758	
				1.54%
		F1-12	1.731	
5	60	F1-13	1.235	2.43%
		F1-14(Centerline)	1.265	
				3.32%
		F1-15	1.307	
6	30	F1-16	0.759	4.08%
		F1-17(Centerline)	0.728	
				1.92%
		F1-18	0.714	
7	45	F1-19	0.975	2.97%
		F1-20(Centerline)	1.004	
				1.69%
		F1-21	0.987	
8	75	F1-22	1.762	2.10%
		F1-23(Centerline)	1.725	
